# Influence of Physical Activity during Pregnancy on Type and Duration of Delivery, and Epidural Use: Systematic Review and Meta-Analysis

**DOI:** 10.3390/jcm12155139

**Published:** 2023-08-05

**Authors:** Dingfeng Zhang, Stephanie-May Ruchat, Cristina Silva-Jose, Javier Gil-Ares, Rubén Barakat, Miguel Sánchez-Polán

**Affiliations:** 1 AFIPE Research Group, Faculty of Physical Activity and Sport Sciences-INEF, Universidad Politécnica de Madrid, 28040 Madrid, Spain; 2 Department of Human Kinetics, Université du Québec à Trois, Trois-Rivières, QC G8T 0A1, Canada

**Keywords:** instrumental delivery, cesarean section, duration of labor, epidural anesthesia, physical activity

## Abstract

Cesarean delivery may increase the need for anesthesia administration, thereby causing potential risks to both maternal and fetal health. This article aimed to investigate the effect of physical activity during pregnancy on the type of delivery, the duration of labor, and the use of epidurals (registration No.: CRD42022370646). Furthermore, 57 RCTs (*n* = 15301) were included showing that physical activity could decrease the risk of cesarean section (z = 3.22, *p* = 0.001; RR = 0.87, 95% CI = 0.79, 0.95, I^2^ = 37%, P_heterogeneity_ = 0.004), and 32 RCTs (*n* = 9468) showed significant decreases in instrumental delivery through performing physical activity (z = 3.48, *p* < 0.001; RR = 0.84, 95% CI = 0.76, 0.93, I^2^ = 0%, P_heterogeneity_ = 0.63). A significant decrease in the 15 RCTs’ (*n* = 4797) duration of first stage labor was found in physically active pregnant women (z = 2.09, *p* = 0.04; MD = −62.26, 95% CI = −120.66, −3.85, I^2^ = 93%, P_heterogeneity_ < 0.001) compared to those not active. Prenatal physical activity could decrease the risk of cesarean section and instrumental delivery and the duration of first stage labor.

## 1. Introduction

Childbirth is a complex process that can have significant implications for the health of both mother and infant. Spontaneous delivery without the need for intervention such as instrumental delivery or cesarean section is recommended [1,2]. However, the World Health Organization (WHO) has stated that the global caesarean section rate has risen from around 7% in 1990 to 21% today. By 2030, it is projected that the caesarean section rate will reach 63% in East Asia, followed by Latin America and the Caribbean (54%), Western Asia (50%), North Africa (48%), Southern Europe (47%), and Australia and New Zealand (45%) [3]. Compared to vaginal delivery, cesarean delivery increases the risk of adverse outcomes for both mother and infant, delays recovery duration, and thus incurs higher medical costs [4]. However, vaginal delivery may also lead to an increase in acute and chronic maternal morbidity [5].

The most common indication for cesarean section is slow progress of labor leading to delayed delivery and maternal fatigue. Prolonged labor can lead to various deleterious consequences for the well-being and health of both the mother and the fetus. For the mother, these include fatigue and physical exhaustion, increased risk of infection, vaginal or perineal injury, and psychological stress. For the fetus, there may be risks of oxygen deprivation, distress, and injury, which can affect the functioning of the brain and other organs [6]. Then, limiting prolonged duration of labor is important. The second stage of labor holds great significance due to its connection with higher rates of maternal and perinatal health issues and even death.

An important breakthrough in delivery management is relieving labor pain. About 80% to 90% of women request and receive epidural anesthesia to alleviate labor pain in United Kingdom hospitals [7]. However, some evidence suggests that while relieving maternal pain, epidural anesthesia also prolongs the second stage of labor, increases the risk of operative delivery [8]. Moreover, this type of anesthesia could directly reduce uterine contraction ability, pelvic floor muscle tension, and reflexive maternal pushing response, all of which are necessary conditions for normal internal rotation of the fetal head [9] and thus for the delivery progress.

It is worth acknowledging that meta-analyses were previously published, showing that engaging in physical activity during pregnancy decreases the odds of cesarean section and instrumental delivery [4,10,11]. Physical activity during pregnancy did not influence the duration of the first stage labor and the use of epidural [12,13]. However, some of the meta-analyses were published five years ago (or even more) and more literature on the topic has been published since. Meta-analyses were published recently (three years ago) with few studies (less than 15 articles); in fact, some important studies have not been included in the analysis. Certainty of previous evidence was not high. Moreover, there were few meta-analyses published about physical activity during pregnancy and duration of labor and use of epidural. Therefore, providing an update of the literature would be important to better understand the impact of prenatal exercise on these outcomes. The objective of this systematic review was to assess the effect of physical activity during pregnancy on the type of delivery, duration of labor, and on the use of epidural anesthesia.

## 2. Materials and Methods

A systematic review was carried out based on the Preferred Reporting Items for Systematic Reviews and Meta-Analyses (PRISMA) guidelines [14]. The protocol was registered in the International Prospective Registry of Systematic Reviews (PROSPERO, registration No. CRD42022370646).

### 2.1. Eligibility Criteria

The eligibility criteria for this systematic review were guided by the PICOS framework: participants, interventions, comparisons, outcomes, and study design [14].

### 2.2. Population

The population of interest was pregnant women without contraindication to exercise (according to the most recent international clinical guidelines about physical activity during pregnancy) [15,16]. Absolute contraindications were defined as: ruptured membranes, premature labor, unexplained persistent vaginal bleeding, placenta previa after 28 weeks’ gestation, pre-eclampsia, incompetent cervix, intrauterine growth restriction, high-order multiple pregnancy (e.g., triplets), uncontrolled type I diabetes, uncontrolled hypertension or uncontrolled thyroid disease, and other serious cardiovascular, respiratory or systemic disorders. Relative contraindications were defined as: recurrent pregnancy loss, history of spontaneous preterm birth, gestational hypertension, symptomatic anemia, malnutrition, eating disorder, twin pregnancy after the 28th week, mild/moderate cardiovascular or respiratory disease, and other significant medical conditions [15,16].

### 2.3. Intervention

Physical activity interventions during pregnancy were searched for. Studies were selected if they reported any type of quantifiable physical activity: frequency, intensity, type and duration of physical activity, duration of the intervention, adherence to the intervention, and mode of delivery of the intervention (supervised or unsupervised physical activity).

### 2.4. Comparison

The comparator was no physical activity (i.e., the control group). Women receiving standard care (i.e., regular obstetrical follow-ups with health care providers) were considered as controls.

### 2.5. Outcome

The primary outcome of interest was the type of delivery (cesarean, instrumental delivery). Secondary outcome was the duration of labor (first, second, and third stage), and the use of epidural anesthesia.

### 2.6. Data Sources

An exhaustive and comprehensive search was carried out using the Universidad Politécnica de Madrid software in the following databases: Academic Search Premier, ERIC, MEDLINE, SPORTDiscus, OpenDissertations, Clinicaltrials.gov, Web of Science, Scopus, and Cochrane Database of Systematic Reviews. To ensure equality in the selection process, the same article selection criteria were used for all databases, considering differences in controlled vocabulary and rules of selection syntax. As articles published in English and Spanish were considered for the search, the search terms used were:

- English: physical activity OR exercise OR training OR physical exercise OR fitness OR strength training OR physical intervention OR Pilates OR Yoga OR strengthening OR aerobic OR resistance training OR pelvic floor muscle training AND pregnancy OR maternal OR antenatal OR pregnant AND type of delivery OR mode of delivery OR duration of labor OR epidural OR anesthetic AND randomized clinical trial OR randomized controlled trial OR RCT OR Quasi experimental clinical trial.
- Spanish: actividad física O ejercicio O entrenamiento O ejercicio físico O fitness O entrenamiento de fuerza O intervención de actividad física O Pilates O Yoga O fortalecimiento O aeróbico O entrenamiento de resistencia O fortalecimiento del suelo pélvico Y embarazo O materno O antenatal O embarazada Y tipo de parto O modo de parto O duración del parto O epidural O anestesia Y ensayo clínico aleatorizado O ensayo controlado aleatorizado O ECA O cuasiexperimental.

### 2.7. Study Selection and Data Extraction

Only randomized controlled trials (RCTs) were selected. Articles published between 2010 and 2023, written in English and Spanish were considered for the search. Reference lists of selected studies, as well as of systematic reviews previously published on the same topic, were retrieved to ensure studies of interest were not missed by the electronic keyword search.

To ensure compliance with the inclusion criteria, two reviewers conducted an independent screening of the titles and abstracts. The abstracts that met the initial screening were then retained for full text revision. The full texts were also revised by two independent reviewers to identify outcomes of interest for data extraction. For studies where one reviewer recommended exclusion and the other inclusion, both reviewers tried to reach a consensus to make a final decision for exclusion or inclusion. In situations of absolute discrepancy, a third reviewer provided their expert opinion on whether the study should be included or excluded.

In cases where a study had multiple publications, the most recent or comprehensive publications was chosen as the primary source. However, relevant data from all the publications were extracted to ensure that no valuable information was overlooked.

Data extraction tables were created in an Excel sheet. One researcher extracted the data and then, data extraction was independently verified by a content expert to facilitate further analysis.

Extracted data were study characteristics (i.e., author last name, year, and country), total sample size and sample size per study group, intervention (type of quantifiable physical activity: frequency, intensity, type and duration of physical activity, duration of the intervention, adherence to the intervention, and supervised or unsupervised physical activity), and primary and secondary outcomes.

### 2.8. Quality of Evidence and Risk of Bias Assessments

To evaluate the certainty of evidence for each study design and outcome, the Grading of Recommendations Assessment, Development, and Evaluation (GRADE) framework was used. This framework provides a standardized and comprehensive approach to assess the certainty of the evidence across multiple studies [17].

To evaluate the risk of bias of RCTs, the Cochrane Handbook was utilized. The potential sources of bias evaluated are: selection bias (inadequate randomization procedures), performances bias (compliance with the intervention), detection bias (flawed outcome measurement), attrition bias (incomplete follow-up and high loss to follow-up), and reporting bias (selective or incomplete outcome reporting) [18].

### 2.9. Statistical Analysis

Statistical analyses were performed with Review Manager software (RevMan, version 5.4). Dichotomous outcomes (i.e., cesarean delivery, instrumental delivery, and the use of epidural anesthesia) were expressed as categorical variable (Yes/No). The number of events in the intervention and control group were recorded and relative risks (RR) and odds ratio (OR) were calculated [19]. For continuous outcomes (duration of first, second, and third stage of labor), mean differences (MD) were calculated [20].

To establish the compensated average in both dichotomous and continuous analyses, a weight system was used that considered the sample size per groups and generally, contributed by each study. A random effects model was used for all analysis. Meta-analyses were performed separately by study design and significance was set at *p*-value < 0.05. To assess the variation in study results between studies (i.e., the degree of heterogeneity), the I^2^ statistic was calculated. The I^2^ statistic was interpreted using established thresholds: low heterogeneity—<25%, moderate heterogeneity—25% to 75%, and high heterogeneity—>75%. In the cases of high heterogeneity, post hoc subgroup analyses were conducted to further explore heterogeneity. In this study we found high heterogeneity with duration of first, second, and third stage labor; therefore, we divided the articles of mentioned analyses into different subgroups according to the age of participants (age ≥ 30 years, 25–30 years, and <25 years).

## 3. Results

**Figure 1:**
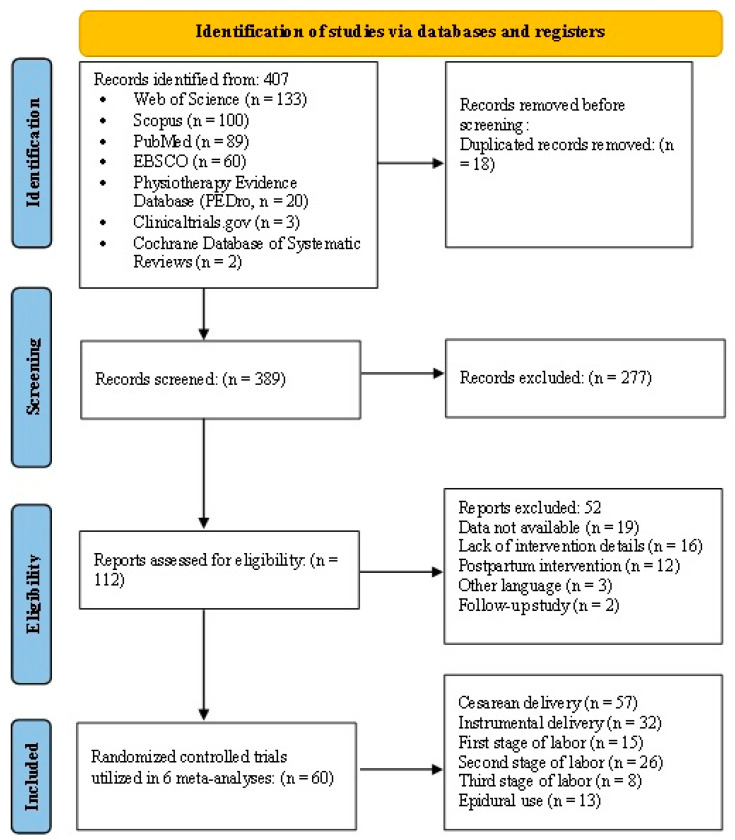
Flow diagram of the selection process.

A total of 60 RCT studies met the inclusion criteria, involving 15,968 pregnant women across 20 countries after the search process that is shown in Figure 1.

Among all of the interventions, 33 included only supervised physical activity, 12 included a combination of supervised and unsupervised physical activity, and 15 included only unsupervised physical activity. Studies varied in frequency of exercise from 1 to 7 days per week, exercise intensity was low to moderate, and the duration of exercise sessions varied between 10 and 75 min. These interventions were carried out during the first, second, or third trimesters, and lasted from 3 to 30 weeks. The type of exercise included walking, stationary cycling, water aerobics, swimming, resistance training, stretching, Pilates, yoga, pelvic floor muscle training, or a combination of various exercise types (Table 1).

### 3.1. Certainty of Evidence and Risk of Bias

**Figure 2:**
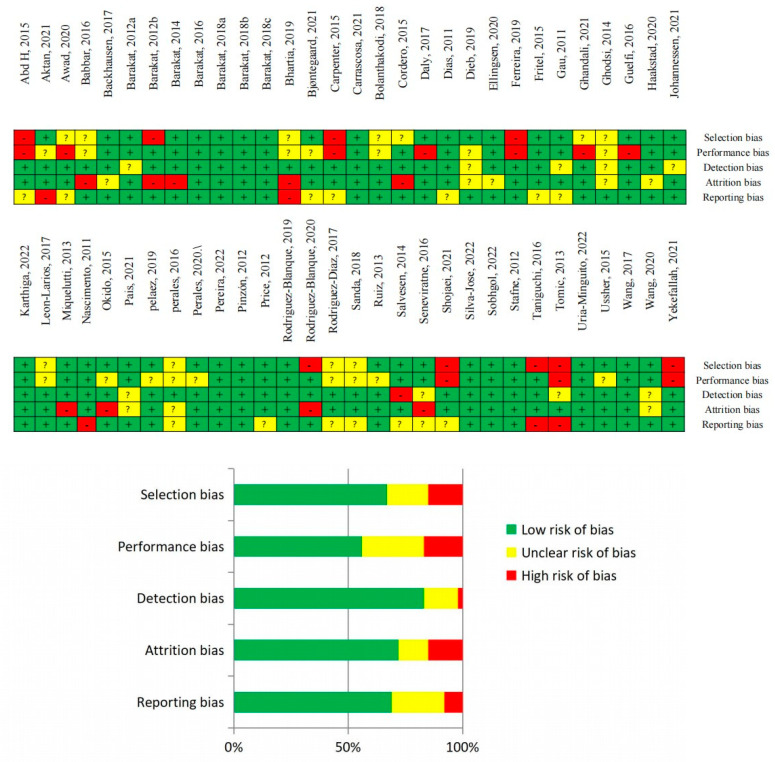
Risk of bias of the included studies [9,21,22,23,24,25,26,27,28,29,30,31,32,33,34,35,36,37,38,39,40,41,42,43,44,45,46,47,48,49,50,51,52,53,54,55,56,57,58,59,60,61,62,63,64,65,66,67,68,69,70,71,72,73,74,75,76,77,78,79].

Collectively, the certainty of evidence was high. In some situations, blinding of participants to the group (intervention or control group) was not feasible, and it is typically impossible to achieve due to the intervention characteristics (physical activity intervention), resulting in unclear or high risk of bias (performance bias) depending on how it was recorded. Other sources of bias found in some cases were the impossibility to find the article protocol published (to compare the planned and measured outcomes), but also not reporting (or being uncertainly defined) the randomization process. Overall, the majority of the studies presented low risk of bias within the five types of bias assessed. Risk of bias analysis is reported in Figure 2.

### 3.2. Effect of Prenatal Physical Activity on Cesarean Delivery

**Figure 3:**
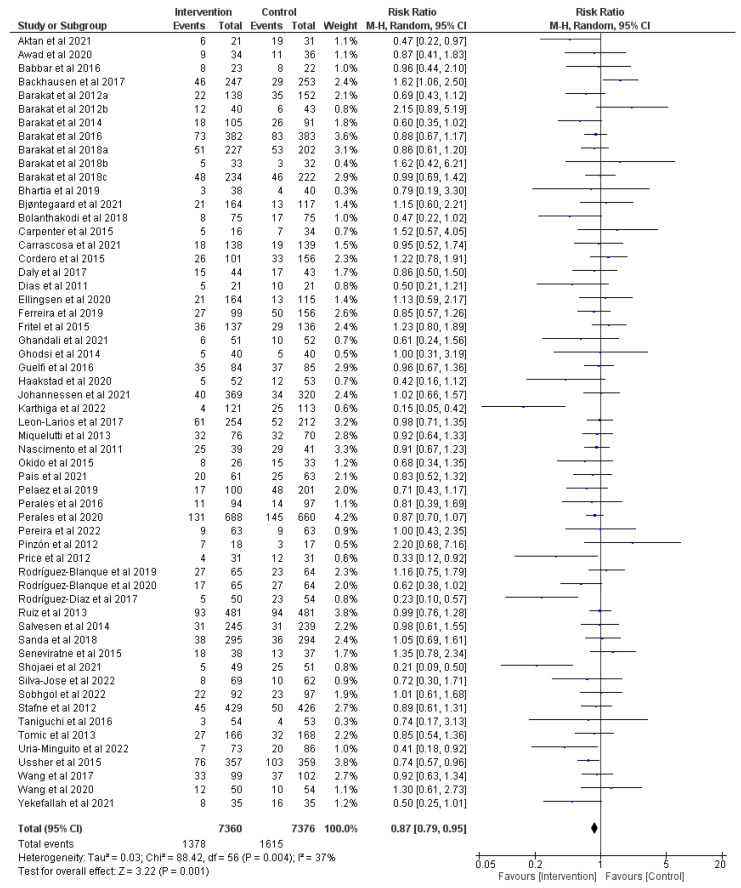
Effect of prenatal physical activity on cesarean delivery [9,22,23,24,25,26,27,28,29,30,31,32,33,34,35,36,37,38,39,41,42,43,45,46,47,48,49,50,51,52,53,54,55,56,57,58,59,60,61,62,63,64,65,66,67,68,69,70,71,72,73,74,75,76,77,78,79].

Overall, there was high certainty of evidence from 57 RCTs (*n* = 15,301) [9,22,23,24,25,26,27,28,29,30,31,32,33,34,35,36,37,38,39,41,42,43,45,46,47,48,49,50,51,52,53,54,55,56,57,58,59,60,61,62,63,64,65,66,67,68,69,70,71,72,73,74,75,76,77,78,79] regarding the effect of prenatal physical activity on cesarean section. A significant decrease in the risk of cesarean deliveries was found with prenatal physical activity compared to no physical activity (z = 3.22, *p* = 0.001; RR = 0.87, 95% CI = 0.79, 0.95, I^2^ = 37%, P_heterogeneity_ = 0.004) as shown in Figure 3.

### 3.3. Effect of Prenatal Physical Activity on Instrumental Delivery

**Figure 4:**
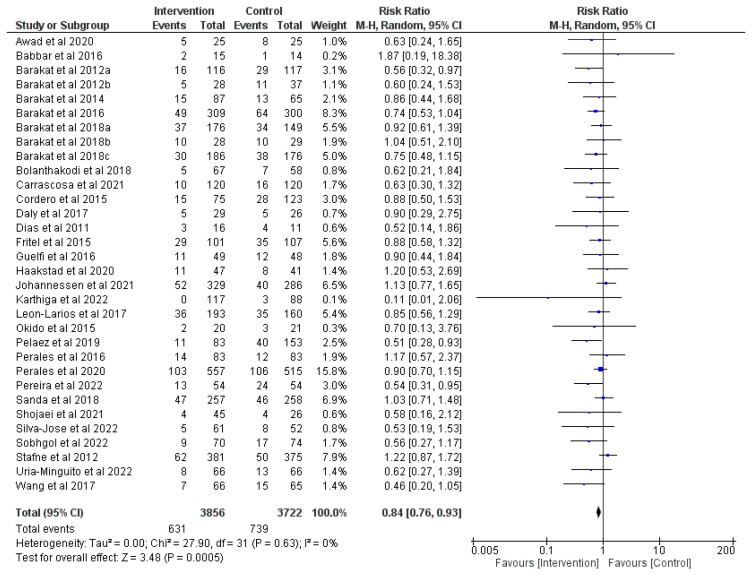
Effect of prenatal physical activity on instrumental delivery [9,23,25,26,27,28,29,30,31,35,36,37,38,39,44,47,48,49,50,51,54,56,57,58,59,67,69,70,71,72,75,77].

In this meta-analysis, 32 RCTs (*n* = 9468) [9,23,25,26,27,28,29,30,31,35,36,37,38,39,44,47,48,49,50,51,54,56,57,58,59,67,69,70,71,72,75,77] reviewing the effect of prenatal physical activity on instrumental section were analyzed. A significant decrease in the risk of instrumental deliveries was found with prenatal physical activity compared to no physical activity (z = 3.48, *p* < 0.001; RR = 0.84, 95% CI = 0.76, 0.93, I^2^ = 0%, P_heterogeneity_ = 0.63). Figure 4 details the current analysis.

### 3.4. Effect of Physical Activity during Pregnancy on Duration of the First Stage of Labor

**Figure 5:**
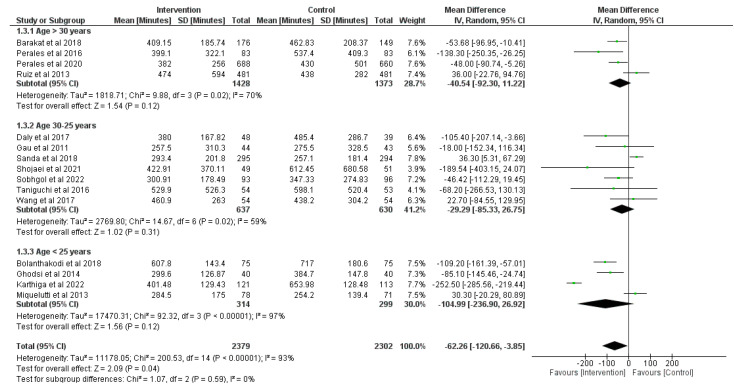
Effect of prenatal physical activity on duration of the first stage of labor [29,36,38,44,46,50,52,57,58,65,67,69,71,73,78].

Fifteen RCTs were analyzed (*n* = 4797) [29,36,38,44,46,50,52,57,58,65,67,69,71,73,78]. Overall, prenatal physical activity was associated with a reduction in the duration of the first stage of labor (in minutes) compared with no physical activity (z = 2.09, *p* = 0.04; MD = −62.26, 95% CI = −120.66, −3.85, I^2^ = 93%, P_heterogeneity_ < 0.001). Due to the high heterogeneity obtained, subgroup analyses were performed to split the studies into three groups depending on the age of participants as is shown in Figure 5. The first subgroup (age > 30 years) shows non-statistical differences between the groups (z = 1.54, *p* = 0.12; MD = −40.54, 95% CI = −92.30, 11.22, I^2^ = 70%, P_heterogeneity_ = 0.02), also not having differences in the second subgroup (age between 25 to 30 years) (z = 1.02, *p* = 0.31; MD = −29.29, 95% CI = −85.33, 26.75, I^2^ = 59%, P_heterogeneity_ = 0.02) and in the third (age < 25 years) group (z = 1.56, *p* = 0.12; MD = −104.99, 95% CI = −236.90, 26.92, I^2^ = 97%, P_heterogeneity_ < 0.001).

### 3.5. Effect of Physical Activity during Pregnancy on Duration of the Second Stage of Labor

**Figure 6:**
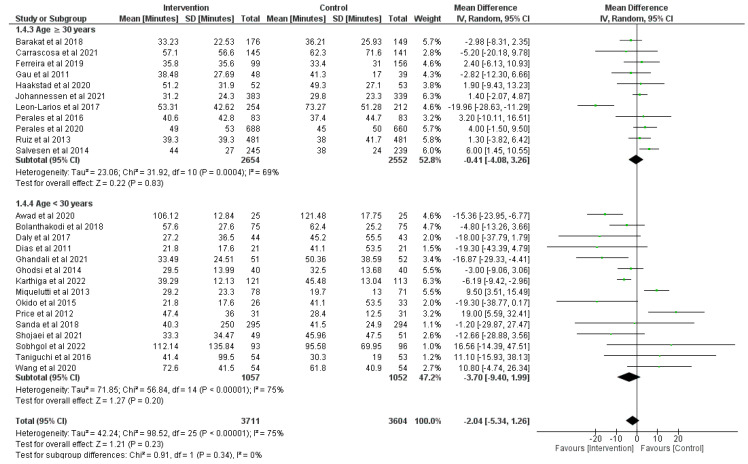
Effect of prenatal physical activity on duration of the second stage of labor [9,29,35,36,38,39,42,44,45,46,47,48,49,50,51,52,54,57,58,61,65,66,67,69,71,73,78].

Overall, there was high certainty of evidence from 26 RCTs (*n* = 7800) [9,29,35,36,38,39,42,44,45,46,47,48,49,50,51,52,54,57,58,61,65,66,67,69,71,73,78] regarding the effect of prenatal physical activity on the second stage of labor. There was no significant decrease in the risk of duration of second stage labor found with prenatal physical activity compared to the non-physical activity group (z = 1.21, *p* = 0.23; MD = −2.04, 95% CI = −5.34, 1.26, I^2^ = 75%, P_heterogeneity_ < 0.001). Subgroup analyses were realized to split the studies into two groups due to high heterogeneity depending on age of participants as is shown in Figure 6. The first subgroup (age ≥ 30 years) shows non-statistical differences between the physical activity group and control group in duration of second stage labor (z = 0.22, *p* = 0.83; MD = −0.41, 95% CI = −4.08, 3.26, I^2^ = 69%, P_heterogeneity_ < 0.001). Similarly, in the second subgroup (age < 30 years) there were no differences between groups in duration of second stage labor (z = 1.27, *p* = 0.20; MD = −3.70, 95% CI = −9.40, 1.99, I^2^ = 75%, P_heterogeneity_ < 0.001).

### 3.6. Effect of Physical Activity during Pregnancy on Duration of the Third Stage of Labor

**Figure 7:**
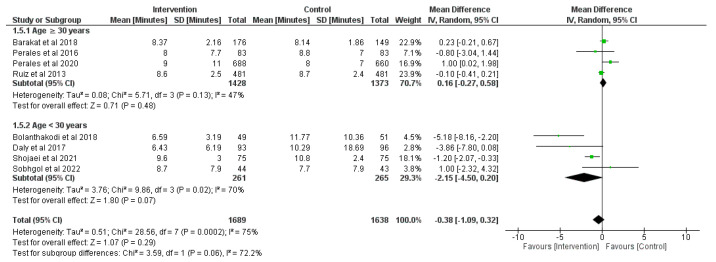
Effect of prenatal physical activity on duration of the third stage of labor [29,36,38,57,58,65,69,71].

Eight RCTs were retrieved and analyzed in this analysis (*n* = 3443) [29,36,38,57,58,65,69,71]. No statistical differences (Figure 7) were observed overall between groups regarding duration of the third stage of labor (z = 1.07, *p* = 0.29; MD = −0.38, 95% CI = −1.09, 0.32, I^2^ = 75%, P_heterogeneity_ < 0.001). It was necessary to split the studies into subgroups due to high heterogeneity present in the general analysis. In the first subgroup no statistical differences were found between study groups (z = 0.71, *p* = 0.48; MD = 0.16, 95% CI = −0.27, 0.58, I^2^ = 47%, P_heterogeneity_ = 0.13). Similarly, in the second subgroup no significant relationships were observable in both groups (z = 1.80, *p* = 0.07; MD = −2.15, 95% CI = −4.50, 0.20, I^2^ = 70%, P_heterogeneity_ = 0.02). 

### 3.7. Effect of Physical Activity during Pregnancy on Epidural Use

**Figure 8:**
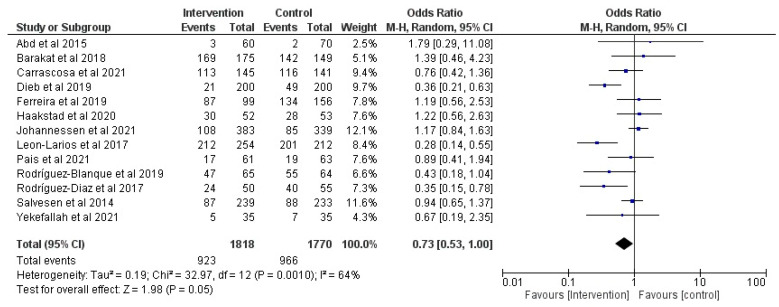
Effect of prenatal physical activity on epidural use [21,29,35,40,42,48,49,51,55,62,64,66,79].

Thirteen RCT articles were analyzed (*n* = 4119) [21,29,35,40,42,48,49,51,55,62,64,66,79] regarding the effect of prenatal physical activity on the use of epidurals. No significant differences were observed in the use of epidural anesthesia between the intervention and control groups (z = 1.98, *p* = 0.05; OR = 0.73, 95% CI = 0.53, 1.00, I^2^ = 64%, P_heterogeneity_ = 0.001) as shown in Figure 8.

## 4. Discussion

In this systematic review, 60 RCTs were included, and there was high certainty of evidence showing that prenatal physical activity could decrease the risk of cesarean delivery by 13% and the risk of instrumental delivery by 16%. We also found that prenatal physical activity was associated with 62.26 min of reduction in the duration of first stage labor.

A review developed by Domenjoz et al. [11] with 16 articles found that women performing exercise during pregnancy had a significant lower risk of cesarean delivery compared to those who did not, and another article published by Wang et al. [12] with 13 RCTs showed that women who exercise during pregnancy had a significantly higher incidence of vaginal delivery than non-physically active women. Furthermore, Davenport et al. [10], in their review published in 2019 with 20 articles analyzed, found that engaging in a prenatal exercise program was associated with a 24% reduction in the likelihood of instrumental delivery. Our systematic review and meta-analysis examined the relationship between physical activity during pregnancy and type of delivery, showing the same conclusions as previously published articles.

Physical activity has been shown to reduce the risk of several pregnancy complications that are often associated with a higher likelihood of cesarean section and instrumental delivery, for example, physical activity during pregnancy has been linked to a lower risk of gestational diabetes mellitus, excessive gestational weight gain, and macrosomia [80,81]. Elsewhere, labor duration is another factor that may contribute to the association between physical activity and decreased risk of cesarean section and instrumental delivery. Regular physical activity during pregnancy has been shown to improve overall fitness, cardiovascular health, and muscle strength [82], which could potentially enhance the efficiency and progress of labor. Shorter labor duration is generally associated with a reduced need for medical intervention, including cesarean section and instrumental delivery. The majority of studies suggest that engaging in regular physical activity during pregnancy can be beneficial for reducing cesarean sections and instrumental deliveries. Consequently, it is necessary that pregnant individuals maintain an active lifestyle throughout their pregnancy.

Our results showed a significant decrease in the duration of first stage labor in the physical activity group compared with the control group. We did not find an association between physical activity during pregnancy and duration of second and third stage labor. Despite the high heterogeneity obtained and after dividing the articles in these three analyses into different subgroups, high heterogeneity was still reported in the first stage of labor meta-analysis (I^2^ = 97%). However, due to the low number of articles and the impossibility to split the articles into other subgroups based on other factors, the research team opted to report the current analysis.

A previously published review [12] found that exercise during pregnancy had no significant influence on first and second stages of labor. Interestingly, a recent review [13] showed that exercise significantly reduced the duration of the second stage of labor, but it did not reduce the first stage of labor, which is contrary to the conclusion drawn from our study. This discrepancy in results between our study and this previous meta-analysis may be attributed to several factors such as differences in study design, inclusion criteria, sample size, or specific characteristics of the populations studied. Regarding the potential link between physical activity and the duration of labor, it is important to note that the first stage of labor is often associated with increased interventions and potential complications. The second stage of labor, although shorter in duration, is crucial for the actual delivery of the baby. The third stage of labor involves the delivery of placenta and is typically shorter compared to the previous stages [83]. Therefore, further research in needed to better understand the potential relationship between physical activity during pregnancy and the different stages of labor.

Our study did not find that prenatal physical activity significantly reducing the need for epidural use during labor. However, it is important to consider the potential advantage of reducing epidural use, such as minimizing medical interventions, promoting a more active and engaged birthing experience, and potentially reducing associated risks or side effects. Further research is essential to better understand the underlying mechanisms and causative factors contributing to this association. Factors such as improved pain tolerance, increased endurance, or enhanced overall fitness may play a role in reducing the need for epidurals among physically activity pregnant women. More researches are needed to better understand the potential importance of incorporating physical activity as part of prenatal care to optimize outcomes and potentially reduce the reliance on epidurals during childbirth.

### Limitations and Strengths

Strengths of this article include the selection and review of both English and Spanish articles expanding the scope of our search in comparison to previous reviews that were restricting to one language, and the inclusion specifically of randomized controlled trials allowing for assessment of the features of physical activity interventions that may not be captured through observational studies (e.g., frequency and type of activity) and are deemed to provide more high certainty of evidence. However, these results should be interpreted with caution in lieu of the inclusion of studies deemed of low quality as well as heterogeneity in the contents of the included interventions. Limitations of this review were the difficulty of obtaining data due to the discrepancies at the moment of reporting data from these studies. This limitation precluded the chance of performing a meta-analysis of the total duration of gestation but also to assess epidural use through a quantitative measure. Other limitations were the high heterogeneity reported in some analyses and the shortage of published articles regarding outcomes of interest as duration of the second stage of delivery. In fact, analyzed articles did not clearly report definitions of stages, possibly increasing variability of analyzed articles per analysis. Division of articles was necessary in three meta-analyses, but due to some retrieved scientific literature not reporting all participant sociodemographic characteristics, it was opted to split articles according to maternal age. Future research should aim to further extrapolate findings based on intensity of the intervention, and types of physical activity.

## 5. Conclusions

This review identified that prenatal physical activity could reduce the risk for cesarean section, instrumental delivery, and decrease the duration of the first stage of labor.

## Figures and Tables

**Table 1:** Characteristics of analyzed articles.

Ref	Country	N	IG	CG	Physical Activity Intervention							Main Variables	Secondary Variables
					Freq	Intens	Durat	Type	Superv	Time	Adh		
Abd et al., 2015 [21]	Egypt	180	110	70	7	Low	10–15	Perineal massage	No	4 w	-	Episiotomy Perineal tear	Type of delivery
							5	Pelvic floor muscle training					
Aktan et al., 2021 [22]	Turkey	43	21	22	2	Mod	60	Clinical Pilates exercise	Yes	8 w	-	General anxiety, gestational weight gain	Type of delivery, birth weight
Awad et al., 2020 [9]	Egypt	50	25	25	3	Mod	60	Aerobic, pelvic floor exercises	Yes	22 w	-	Duration of the second stage labor	Type of delivery and Apgar scores
					3		35		No				
Babbar et al., 2016 [23]	USA	46	23	23	3	Mod	60	Yoga	Yes	8 w	80%	Umbilical artery, type of delivery, birth weight	Gestational weight gain
Backhausen et al., 2017 [24]	Denmark	516	258	258	2	Low	70	Water exercises	No	12 w	76%	Low back pain, birth weight	Type of delivery
Barakat et al., 2012 [25]	Spain	290	138	152	3	Mod	40–45	Aerobic exercise	Yes	28 w	-	Type of delivery	Gestational weight gain birth weight
Barakat et al., 2012 [26]	Spain	83	40	43	3	Low-Mod	35–45	Land aerobic and aquatic activity	Yes	28 w	-	Gestational weight gain and gestational diabetes	Gestational age, type of delivery, birth weight and Apgar score
Barakat et al., 2014 [27]	Spain	200	107	93	3	Low-Mod	55–60	Aerobic exercise, pelvic floor muscle training	Yes	28 w	80%	Gestational age, gestational weight gain, type of delivery, gestational diabetes	Birth weight, head circumference
Barakat et al., 2016 [28]	Spain	765	382	383	3	Mod	50–55	Aerobic, strength, and flexibility exercise	Yes	28 w	80%	Hypertension	Type of delivery, gestational weight gain, birth weight
Barakat et al., 2018 [29]	Spain	429	227	202	3	Mod	55–60	Aerobic exercise	Yes	28 w	80%	Duration of labor	Type of delivery, use of epidural, birth weight
Barakat et al., 2018 [30]	Spain	65	33	32	3	Mod	55–60	Aerobic, pelvic floor strength, and flexibility exercises	Yes	28 w	85%	Placenta weight	Gestational age, type of delivery, birth weight
Barakat et al., 2018 [31]	Spain	456	234	222	3	Mod	50–55	Aerobic exercise	Yes	28 w	-	Gestational weight gain	Gestational age, type of delivery, birth weight
Bhartia et al., 2019 [32]	India	78	38	40	1	Mod	50	Yoga	Yes	12 w	-	Maternal Stress, type of delivery, birth weight	-
					2				No				
Bjøntegaard et al., 2021 [33]	Norway	281	164	117	1	Mod-High	60	Aerobic, strength training and balance exercises	Yes	12 w	-	Type of delivery, birth weight	Physical activity of children at age of seven
					2		45		No				
Bolanthakodi et al., 2018 [34]	India	150	75	75	3	Mod	30	Yoga	No	9 w	-	Pain intensity, type of delivery, duration of delivery	Low birth weight, Preterm birth
Carpenter et al., 2015 [35]	UK	50	16	34	1	Low-Mod	40	Stationary cycling, pelvic floor exercises and water exercises	Yes	18 w	-	Hemodynamic function	Type of delivery, birth weight
Carrascosa et al., 2021 [36]	Spain	286	145	141	3	Mod	45	Aquatic aerobic exercise	Yes	20 w	-	Use of epidural analgesia during labor	Type of delivery, time of active labor, episiotomy
Cordero et al., 2015 [37]	Spain	257	101	156	1–2	Low	50–60	Aerobics in gym hall and aquatic activity	Yes	26 w	80%	Gestational Diabetes	Gestational weight gain, type of delivery, birth weight
Daly et al., 2017 [38]	Ireland	88	44	44	3	Mod	50–60	Aerobic, pelvic floor exercises	Yes	26 w	-	Maternal fasting plasma glucose	Type of delivery, duration of labor, birth weight
Dias et al., 2011 [39]	Norway	42	21	21	1	Low	30	Pelvic floor muscle training	Yes	16 w	75%	Type of delivery, duration of labor, birth weight	Pelvic floor muscle strength
					6				No				
Dieb et al., 2019 [40]	Egypt	400	200	200	3	Low	5	Pelvic floor muscle training	No	4 w	-	Episiotomy, perineal tear, type of delivery	Duration of labor, fetal distress, episiotomy, birth weight
					3		10						
Ellingsen et al., 2020 [41]	Norway	279	164	115	1	Mod	60	Aerobic and strength exercises	Yes	12 w	-	Neurodevelopmental in 7-year-old children	Gestational age, birth weight, type of delivery
					2		45		No				
Ferreira et al., 2019 [42]	Portugal	255	99	156	3	Mod	45–60	Aerobic, strength, coordination and flexibility exercises	Yes	24 w	-	Duration of labor, type of delivery	Episiotomy, perineal tear
Fritel et al., 2015 [43]	France	282	140	142	1	Low	20–30	Pelvic floor training	Yes	8 w	-	Urinary incontinence	Type of delivery, birth weight
Gau et al., 2011 [44]	China	87	48	39	3	Low	20	Ball exercise	No	8 w	-	Childbirth pain	Duration of labor
Ghandali et al., 2021 [45]	Iran	103	51	52	2	Low-Mod	35	Pilates exercise	Yes	8	-	Type of delivery, episiotomy, duration of labor	Maternal satisfaction with childbirth process
Ghodsi et al., 2014 [46]	Iran	80	40	40	3	Low	15	Stationary cycling	No	15 w	-	Gestational weight gain, type of delivery, perineal tear	Pregnancy length, first and second stage of labor, Apgar score
Guelfi et al., 2016 [47]	Australia	172	85	87	3	Mod	20–60	Stationary cycling program	Yes	14 w	-	Gestational diabetes	Type of delivery, birth weight
Haakstad et al., 2020 [48]	Norway	105	52	53	2	Mod	60	Aerobic dance and strength training	Yes	12 w	80%	Birth weight	Gestational age, type of delivery
					1		30		No				
Johannessen et al., 2021 [49]	Norway	722	383	339	1	Mod	55–70	Aerobic, strength and pelvic floor exercises	Yes	12 w	-	Urinary incontinence at 3 months postpartum	Type of delivery, episiotomy, epidural, duration of labor, birth weight
					2		45		No				
Karthiga et al., 2022 [50]	India	234	121	113	7	Mod	60	Yoga	No	20 w	-	Gestational hypertension, preeclampsia, premature delivery	Type of delivery, duration of labor, birth weight
León-Larios et al., 2017 [51]	Spain	466	254	212	5	Low	18–23	Perineal massage and pelvic floor exercises	No	6 w	-	Perineal tear and episiotomy	Type of delivery, duration of labor, birth weight and epidural
Miquelutti et al., 2013 [52]	Brazil	149	78	71	7	Low	10–30	Aerobic and pelvic floor muscle exercises	No	14 w	-	Urinary incontinence, lumbopelvic pain and anxiety	Type of delivery, duration of labor
Nascimento et al., 2011 [53]	Brazil	80	39	41	1	Low-Mod	40	Aerobic exercise and walking	Yes	17 w	62.5%	Scoring women on meeting the intervention goals	Gestational weight gain, birth weight, macrosomia, and low birth weight
					5				No				
Okido et al., 2015 [54]	Brazil	59	26	33	7	Low	20	Pelvic floor muscle training	No	16 w	-	PI of the uterine artery, type of delivery, duration of delivery, birth weight	Episiotomy, urinary incontinence
Pais et al., 2021 [55]	India	124	61	63	7	Low	45	Yoga	No	20 w	-	Preeclampsia and gestational diabetes	Gestational age, duration of labor, type of delivery, birth weight
Pelaez et al., 2019 [56]	Spain	345	230	115	3	Low-Mod	60–65	Aerobic and resistance training	Yes	24 w	80%	Gestational weight gain	Gestational diabetes, macrosomia, type of delivery
Perales et al., 2016 [57]	Spain	166	83	83	3	Low-Mod	55–60	Aerobic, strength exercises, pelvic floor muscle training	Yes	28 w	-	Duration of labor, gestational age, gestational weight gain, type of delivery, birth weigh	Birth size, head circumference, Apgar score
Perales et al., 2020 [58]	Spain	1348	668	660	3	Low-Mod	50–55	Aerobic and pelvic floor exercises	Yes	30	95%	Gestational weight gain, hypertension and diabetes	Type of delivery, birth weight, gestational age
Pereira et al., 2022 [59]	Portugal	126	63	63	3	Low-Mod	30	Walking	Yes	3 w	-	Rate of labor induction	Type of delivery, birth weight
Pinzón et al., 2012 [60]	Colombia	64	31	33	3	Low-Mod	60	Aerobic and stretching exercises	Yes	12 w	-	Gestational age, gestational weight gain, type of delivery	Birth weight, birth size, head circumference, Apgar score
Price et al., 2012 [61]	USA	62	31	31	3	Mod	45–60	Aerobic exercise and walk briskly	Yes	23 w	-	Gestational weight gain. duration of labor, birth weight, postpartum recovery	Length of first and second stage of labor, type of delivery, gestational diabetes
					1		30–60		No				
Rodríguez-Blanque et al., 2019 [62]	Spain	129	65	64	3	Mod	60	Aquatic physical exercise	Yes	17 w	-	Laceration and episiotomy rates	Type of delivery, birth weight and anesthesia
Rodríguez-Blanque et al., 2020 [63]	Spain	129	65	64	3	Mod	60	Aquatic physical exercise	Yes	17 w	-	Gestational weight gain, type of delivery	Birth weight, Apgar score
Rodríguez-Diaz et al., 2017 [64]	Spain	100	50	50	2	Mod	40–45	Pilates	Yes	8 w	90%	Gestational weight gain, blood pressure, strength, flexibility, and spinal curvature	Type of delivery, episiotomy analgesia and birth weight
Ruiz et al., 2013 [65]	Spain	962	481	481	3	Low-Mod	50–55	Aerobic and resistance exercises	Yes	28 w	97%	Gestational weight gain	Birth weight, duration of labor
Salvesen et al., 2014 [66]	Sweden	855	427	426	1	Low-Mod	55–70	Aerobic, strength and pelvic floor exercise	Yes	12 w	-	Gestational diabetes	Urinary and anal incontinence, lumbopelvic pain, and duration of labor
					2		45		No				
Sanda et al., 2018 [67]	Norway	589	295	294	3	Mod	60	Aerobic exercises	Yes	22 w	-	Gestational age, duration of labor, type of delivery	-
					2		30		No				
Seneviratne et al., 2015 [68]	New Zealand	75	38	37	3–5	Mod	15–30	Stationary cycling program	No	16 w	33%	Birth weight, type of delivery	Gestational weight gain, gestational age
Shojaei et al., 2021 [69]	Iran	100	49	51	4	Mod	40	Walking	No	4 w	-	Duration of labor	-
Silva-Jose et al., 2022 [70]	Spain	157	78	79	3	Mod	55–60	Aerobic, strength and pelvic floor exercises	Yes	28 w	80%	Gestational weight gain	Type of delivery, birth weight
Sobhgol et al., 2022 [71]	Australia	200	100	100	1–2	Low	10	Pelvic floor muscle exercises	No	16 w	50%	Female Sexual Function	Type of delivery, perineal tear, episiotomy, duration of labor, birth weight
Stafne et al., 2012 [72]	Norway	761	396	365	1	Mod-High	60	Aerobic, strength, and pelvic floor exercises	Yes	12 w	-	Gestational diabetes, insulin resistance	Birth weight, gestational age, Apgar scores
					3		45		No				
Taniguchi et al., 2016 [73]	Japan	118	60	58	3	Mod	30	Walk briskly	Yes	6 w	80%	Duration of labor; type of delivery, birth weight	-
Tomic et al., 2013 [74]	Croatia	334	166	168	3	Low-Mod	50	Aerobic exercise	Yes	28 w	80%	Macrosomia birth weight, gestational weight gain	Preeclampsia, gestational diabetes, type of delivery
Uria-Minguito et al., 2022 [75]	Spain	203	102	101	3	Mod	50–60	Aerobic, strength, and pelvic floor exercises	Yes	28 w	-	Gestational diabetes	Gestational weight gain, type of delivery, birth weight
Ussher et al., 2015 [76]	UK	789	394	395	3–4	Low	20	Exercise on a treadmill	Yes	6 w	-	Continuous smoking abstinence	Gestational age, preterm birth, type of delivery, birth weight
Wang et al., 2017 [77]	China	226	112	114	3	Mod	45–60	Stationary cycling program	Yes	24 w	75%	Gestational diabetes	Gestational weight gain, birth weight, macrosomia
Wang et.al., 2020 [78]	China	108	54	54	7	Low	30	Pelvic floor muscle training	No	12 w	-	Stress urinary incontinence, episiotomy	Duration of labor and type of delivery
Yekefallah et al., 2021 [79]	Iran	70	35	35	2	Low -Mod	75	Yoga	Yes	11 w	-	Episiotomy, perineal tear, type of delivery	Birth weight, gestational age, duration of labor

IG, intervention group; CG, control group; Freq, weekly frequency; Intens, intensity; Mod, moderate; Durat, minutes of session duration; Time, weeks of intervention; Sup, supervised sessions.

## Data Availability

The data presented in this study are available on request from the corresponding author.
